# Anxiety and Depressive Disorders and Quality of Life Assessment of Poles—A Study Covering Two Waves of the COVID-19 Pandemic

**DOI:** 10.3389/fpsyt.2021.704248

**Published:** 2021-10-22

**Authors:** Mateusz Babicki, Bogna Bogudzińska, Krzysztof Kowalski, Agnieszka Mastalerz-Migas

**Affiliations:** ^1^Department of Family Medicine, Wroclaw Medical University, Wroclaw, Poland; ^2^Students' Scientific Group at the Faculty of Psychiatry, Wroclaw Medical University, Wroclaw, Poland

**Keywords:** COVID-19, anxiety, depression, quality of life, mental health

## Abstract

**Background:** More than a year after the first case of SARS-CoV-2 (Severe Acute Respiratory Syndrome Coronavirus-2) viral pneumonia, the world is still engulfed by the pandemic, and we know that this condition has an enormous impact not only on individuals but also on the social order in virtually every aspect of daily life, deteriorating our mental health. This study aims to assess the prevalence of depressive and anxiety symptoms and the subjective assessment of the quality of life in the different stages of the COVID-19 (Coronavirus Disease 2019) pandemic based on a nationwide online survey.

**Materials and Methods:** The study was conducted using an original questionnaire assessing the sociodemographic status and standardized psychometric tools: Beck Depression Inventory (BDI), Generalized Anxiety Disorder (GAD-7) and Manchester Short Assessment of Quality of Life (MANSA). The study was conducted in two stages corresponding to the first and second wave of the COVID-19 pandemic.

**Results:** In total, 4,083 respondents participated in the survey. The first observation stage took place between 17 and 26 April 2020 and comprised 2,457 respondents; the repeated survey that took place between 1 and 30 December 2020 comprised 1,626 respondents. In both cases, women constituted the majority of respondents (82.5% in the first stage and 79.6% in the second stage). Statistically significantly higher levels of depression and anxiety were found in second stage, with mean scores of BDI and GAD-7. In the case of MANSA, participants in the different stages of the pandemic showed no significant differences in terms of mean scores. However, women were more susceptible to developing the depression and anxiety symptoms and it was obtained in both waves of the pandemic

**Conclusions:** As the Covid-19 pandemic progressed, there was higher level of depressive and anxiety symptoms among Poles.

## Introduction

More than a year after the first case of SARS-CoV-2 viral pneumonia, the world is still engulfed by the pandemic, which was officially declared on 11 March 2020 by the WHO, with many countries fighting its successive waves (1). We know that the pandemic has a considerable impact on individuals but also entire societies, changing many aspects of daily life (2). As time passes, the negative effects on social life and mental health escalate (3). Long-lasting restrictions, together with their periodic tightening, lead to a worsening economic situation so that an increasing number of people lose their jobs or their earning opportunities become significantly limited (4). Economic uncertainty is considered one of the main stressors (5). Many authors point out that a similar psychological discomfort may be related to the anxiety about one's health, which in addition to the threat of potential SARS-CoV-2 infection may be partly caused by limitations in the functioning of the healthcare system (6). On the other hand, it is worth noting that due to that limitations, telemedicine and devices measuring life parameters 24 h a day developed rapidly (7). Some studies show that this contributed to reducing anxiety and a sense of isolation among patients (8). The vast majority of the healthcare system is focused on the care of COVID-19 patients (9). Apart from the number of deaths directly related to COVID-19, the population-based mortality rate in many European Union countries, such as Poland, Spain and Italy, also increased dramatically during the peaks of COVID-19 cases in those countries compared to similar periods in previous years. This shows what a difficult time the European health sectors are currently facing. A recent Eurostat study shows that during the peak of the COVID-19 pandemic in Poland, mortality increased by 97.5%, putting Poland at the top of that statistic (10).

The experience of the previous SARS pandemic clearly supports the fact that the changes to date and the effects are likely to have a significant impact on people's psyche and their quality of life in the long term, and this process should be monitored continuously so that appropriate prevention programmes can be implemented (11). The literature review to date shows that COVID-19 will also have an impact on the condition of people. Studies conducted in many countries show its impact in the initial stage, and as it progresses, the impact on indirect factors, including the course, place, level, and the past effect of psychiatric treatment. It is also very much related to factors regarding the variables in a country: the number of cases, deaths and the ongoing restrictions. Isolation and the feeling of insufficient social support may contribute to the deterioration of the mental condition of citizens (12, 13). Limiting interpersonal contacts and satisfying activities, as a result of long-term restrictions, may cause the development of apathy, sadness, anxiety, depression, and insomnia. Loneliness, which worsens in a pandemic, may be related to the emergence of suicidal thoughts (12). According to some scientific reports, the second wave of the COVID-19 pandemic exacerbated the above-mentioned symptoms (14, 15). The research conducted in Japan on a group of 2,708 people using the PHQ-9, STAXI, Brief Cope scale confirms the severity of depressive symptoms in the general population. It was confirmed that marital status and employment have a stronger influence on mental health than stress coping strategies (14). Similar observations from Poland with the use of the HADS, PSS10, COPE, Audit, SDA, FCV-19S scales confirmed the intensification of depression and anxiety symptoms and the intensification of suicidal thoughts among the respondents (15). Moreover, it has been shown that women are more prone to escalation of depression and anxiety, which may result from their greater sensitivity to stress. The deterioration of the socio-economic situation also contributes to the deterioration of the mental condition. It is often associated with adversities such as losing a job, falling household income, and having difficulty paying bills (16–19).

According to observations from around the world, also in Poland, studies from the beginning of the pandemic indicate a deterioration of the mental condition to the state before the pandemic (12–16). Therefore, to understand the long-term impact of the COVID-19 pandemic on the psychological well-being of people, it is necessary to constantly monitor it, taking into account both its waves and the period between the increase in disease and easing the prevailing restrictions. This is due to the differences in the individual stages of the pandemic, which may include the level of morbidity, mortality, the type of restrictions introduced and, most importantly, the approach of people to the prevailing epidemiological situation. According to the authors' knowledge, this is the first study conducted in Poland to assess the severity of anxiety, depression and quality of life disorders in individual waves of the COVID-19 pandemic.

Given the above, this study aims to assess the prevalence of depressive and anxiety symptoms and the subjective assessment of the quality of life in the different stages of the COVID-19 pandemic based on a nationwide online survey. To this end, we posed the following research hypotheses which were formulated based on previous experiences and other scientific reports: (1) As the COVID-19 pandemic continues, a high number of individuals with depressive and anxiety disorders will be observed and quality of life will be decreased. (2) There is a close relationship between the severity of anxiety and depressive symptoms and the subjective assessment of the quality of life. (3) Women, residents of large cities, and those with a psychiatric history will exhibit poorer mental health. (4) Economic instability significantly affects mental health.

## Materials and Methods

### Methodology

A two-stage CAWI (Computer-Assisted Web Interview) survey was conducted using an original, fully anonymous and voluntary questionnaire distributed online via a social media portal. The questionnaire was addressed to persons over 18 with access to the Internet who were staying in Poland at the time of the survey. The first stage of observation covered the initial period of the COVID-19 pandemic (17–26 April 2020, i.e., a month after the first case of SARS-CoV-2 infection was diagnosed in Poland), when the daily increase in new COVID-19 infections oscillated between 263 and 460 cases, and the number of deaths ranged from 18 to 40 (20). At that time, restrictions in place included the closure of schools, shops excluding groceries, theaters, cinemas, swimming pools, gyms, restaurants (takeaway food could be ordered), hairdressing and hotel services (21). The questionnaire was redistributed during some of the highest incidence and death rates in Poland (1–30 December 2020), ranging from 2,921 to 14,835 and 29 to 620, respectively (22). Due to the deteriorating epidemiological situation, the government decided to return, after the holiday loosening of restrictions, to some of the preventive measures from the beginning of the pandemic, which included the closure of schools, theaters, cinemas, swimming pools, gyms, restaurants (takeaway food could still be ordered) and hotel services. Compared to the first stage, shopping centers and hair and beauty salons remained open (23).

The respondents were informed about the study objectives, methodology, and estimated duration before participating. During its course, the respondents were allowed to withdraw from it without giving any reason. After reviewing the information, they gave informed consent to participate in the study by approving the subpage of the questionnaire. The Bioethics Committee of the Wroclaw Medical University approved the study; it was conducted in line with the Declaration of Helsinki. The original questionnaire contained closed, single-choice questions. It included items assessing sociodemographic status, including age, sex, place of residence, level of education, occupation and limitation of earning opportunities due to the COVID-19 pandemic. Personal experiences of mental illness (own illness, loved one's illness, and pharmacotherapy) and COVID-19 (suspected, confirmed illness, quarantine, or isolation) were also assessed. Subjective feelings of anxiety [evaluated using a linear scale from 1 (no anxiety) to 10 (extreme anxiety)] about contracting COVID-19, anxiety about a neighbor in quarantine or their illness, and the level of adherence to government recommendations to stop the spread of SARS-CoV-2 were also assessed. The level of fear of the disease was also compared in relation to other diseases with the question “Are you afraid of falling ill with COVID-19?” with possible answers: Yes, to the same extent as for other diseases (e.g., heart diseases)/Yes, but to a lesser extent than for other diseases (e.g., heart diseases) / No, I'm not afraid at all.

The next part of the questionnaire consisted of standardized psychometric tools:

a) **The Generalized Anxiety Disorder (GAD-7)** scale is a seven-item psychometric tool for assessing anxiety levels and the risk of developing generalized anxiety. The questionnaire includes seven items, based on a four-point Likert scale, which allow respondents to determine feelings of anxiety, tension, nervousness, the ability to control these feelings, the ease with which they arise and problems with relaxation. For each question, the respondents could score from 0 to 3 points, depending on the frequency with which a given phenomenon occurred (0—not at all; 1—several days; 2—more than half days; 3—nearly every day) within 14 days before the survey. The cut-off point for the occurrence of anxiety was 5 points. A score of 5, 10, and 15 points indicates mild, moderate and severe anxiety, respectively. A score of at least 10 points indicates a high probability of generalized anxiety disorder (24).
b) **The Beck Depression Inventory** is the most commonly used tool for measuring the depth of depression. It consists of 21 questions in which the respondents assess the intensity of symptoms on a scale from 0 to 3 points, most accurately describing their mood over the past 14 days. The following cut-off points are used to interpret the results: 0–9 points: no depression; 10–19: mild depression; 20–25: moderate depression; 26–63: severe depression (25, 26).
c) **The Manchester Short Assessment of Quality of Life (MANSA)** is a 16-item scale for subjective evaluation of respondents' own lives and various aspects thereof. It is based mainly on a seven-point Likert scale (apart from questions concerning the confirmation or negation of a given phenomenon), where the answers were assigned the corresponding score (1 = could not be worse; 2 = I am dissatisfied; 3 = I am somewhat dissatisfied; 4 = I have mixed feelings; 5 = I am somewhat satisfied; 6 = I am satisfied; 7 = it could not be better). The maximum number of points on the test is 93. The tool was developed based on the Lancashire Quality of Life Profile (LQLP), constituting its condensed alternative while maintaining the psychometric parameters (27, 28). The questionnaire was designed for population studies without precise determination of the quality of life assessment in particular diseases.

### Statistical Analysis

The statistical analysis was conducted using Statistica software, version 13.3 (StatSoft). Basic descriptive statistics methods were applied to the quantitative variables. A chi-squared test was used for determining the relationships between the compared ordinal variables. The normality of the distribution was determined using the Kolmogorov-Smirnov, Lilliefors and Shapiro-Wilk W tests. If the distribution was not normal, the U-Mann-Whitney or Kruskal-Wallis test was used. A comparison was made between the score of the GAD-7 and the subjective feelings of anxiety concerning the respondents' own illness and the illness of their loved ones and the neighbor's quarantine in relation to individual stages of the survey. Similarly, a comparative analysis was carried out between the scores of the Beck Depression Inventory and the Manchester Short Assessment of Quality of Life. The effect of sociodemographic factors such as sex, place of residence, education level, relationship status and individual experience with mental illness and COVID-19 of respondents and their loved ones on the GAD-7 scale, Beck Depression Index and the Manchester Short Assessment of Quality of Life in each stage of the survey was assessed. Spearman's correlation analysis was performed between scales and age for both stages of the study.

An analysis of covariance (ANCOVA) was carried out to detect the effect of potential confounding factors on differences between study stages in assessing the GAD-7, BDI, and MANSA. A statistical significance level of > 0.05 was assumed at each stage of the survey.

## Results

### Participants

A detailed description of the study group is presented in Table 1.

**Table 1:** Characteristics of the study group by study stage and the results of analyses comparing the two assessments.

**Variable**	**Stage 1 (*n =* 2,457) *N* (%)**	**Stage 2 (*n =* 1,626) *N* (%)**	**χ^2^**	Effect size^**^	*p* ^*^
**Sex**					
Female	2,027 (82.5)	1,294 (79.6)	5.485	0,036	0.191
Male	430 (17.5)	332 (20.4)			
**Place of residence**					
Rural area	463 (18.8)	287 (17.6)	0.929	0,015	0.336
Town/city	1,994 (81.2)	1,339 (82.4)			
**Education**					
University education	1,987 (80.9)	1,158 (71.2)	51.526	0,112	** <0.001**
Other	470 (19.1)	468 (28.8)			
**Relationship status**					
In a relationship	1,427 (58.1)	609 (37.4)	166.494	0,241	** <0.001**
Single	1,030 (41.9)	1,017 (62.6)			
**Past psychiatric treatment**					
Yes	510 (20.8)	333 (20.5)	0.046	0,003	0.830
No	1,947 (79.2)	1,293 (79.5)			
**Recent suspicion of Covid-19**					
Yes	78 (3.2)	322 (19.8)	306.164	0,273	** <0.001**
No	2,379 (96.8)	1,304 (80.2)			
**Recent mandatory quarantine**					
Yes	84 (3.4)	271 (16.7)	228.471	0,230	** <0.001**
No	2,373 (96.6)	1,355 (83.3)			
**COVID-19 diagnosis**					
Yes	5 (0.2)	138 (8.5)	198.659	0,221	** <0.001**
No	2,452 (99.8)	1,488 (91.5)			
**COVID-19 diagnosis in loved ones**					
Yes	119 (4.8)	1,035 (63.6)	1,669.090	0,639	** <0.001**
No	2,338 (95.2)	591 (36.4)			
**Search for additional information on Covid-19, e.g., online**					
Yes	1,529 (62.2)	775 (47.7)	84.451	0,143	** <0.001**
No	928 (37.8)	851 (52.3)			
**Daily tracking of statistics on behavior and mortality due to COVID-19**					
Yes	1,562 (63.6)	781 (48.0)	96.642	0,153	** <0.001**
No	895 (36.4)	845 (52.0)			
**Loss of earning opportunities due to the pandemic**					
Yes	604 (24.6)	340 (20.9)	7.424	0,042	**0.006**
No	1,853 (75.4)	1,286 (79.1)			

* *χ^2^ test*.

** *Fi/Cramer's V*.

*Significant effects (p < 0.05) are marked in bold*.

The survey comprised 4,083 respondents. The first stage included 2,457 respondents, of which 82.50% were female, and the mean age was 32 years (min. 18, max. 83; SD 10.67). For the next stage, 1,626 participants were recruited with a female predominance of 79.6%, and the mean age was 24 years (min. 18, max. 78; SD 7.06). In both stages, the vast majority were people living in cities with higher education. In addition, in the second stage more people had a diagnosis of COVID-19 (or a family member) and were forced to quarantine. On the other hand, far fewer were looking for additional information on COVID-19 or browsing statistics on behavior and mortality due to COVID-19.

### Perception of Anxiety (GAD-7) and Compliance With Government Recommendations

A detailed analysis of the GAD-7 and individual linear scales is presented in Table 2.

**Table 2:** Analysis of the GAD-7 and the COVID-19 subjective feeling of anxiety scale concerning oneself, neighbors and their quarantine and adherence to government recommendations.

**GAD-7 interpretation**	**Stage 1 (*n =* 2,457) *N* (%)**	**Stage 2 (*n =* 1,626) *N* (%)**	**χ^2^**	Effect size^***^	***p***
Mean score	9.11 ± 6.17	9.52 ± 6.04		−0.067	0.022^*^
No anxiety	709 (28.9)	423 (26.0)	5.896	0.038	0.112^**^
Mild anxiety	660 (26.9)	424 (26.1)			
Moderate anxiety	518 (21.1)	372 (22.9)			
Severe anxiety	570 (23.2)	407 (25.0)			
**COVID-19 subjective feeling**					
Anxiety over getting COVID-19	5.50 ± 2.63	4.86 ± 2.45		0.251	<0.001^*^
Anxiety due to neighbors in quarantine	4.62 ± 2.77	3.02 ± 2.33		0.625	<0.001^*^
Anxiety due to neighbors getting COVID-19	5.73 ± 3.01	3.63 ± 2.63		0.742	<0.001^*^
Adherence to government recommendations	8.67 ± 1.65	7.63 ± 1.99		0.568	<0.001^*^

* *U-Mann Whitney test*.

** *χ^2^ test*.

*** *d Cohen for U-Mann Whitney test and Fi/Cramer's V for χ^2^ test*.

In the first stage of the survey, 87.4% of respondents admitted to being anxious about contracting COVID-19, with 26.5% being more anxious over COVID-19 and 23.2% less afraid of COVID-19 than other conditions such as heart disease. In the repeat survey, there was a lower level of subjective feelings of anxiety and SARS-CoV-2 infection was feared by 80.7% of respondents, while 13.7% were more anxious about the infection than other somatic conditions. 33.9% of respondents reported anxiety over COVID-19 but to a lesser extent than other conditions.

In the overall analysis of the GAD-7 scale, a statistically significantly higher mean score was found in the second stage of the study, but the effect size was very small. There was no association between presenting anxiety disorder and survey waves.

Linear scale analysis (1–10 points) of subjective feelings of anxiety about contracting COVID-19 and neighbors being in quarantine or ill showed a significant difference in the later stage of the pandemic compared to its initial stage. There was also a difference in the public's compliance with the official recommendations to combat COVID-19, measured on a scale of 1 to 10 points.

### Depressive Symptoms (BDI)

A detailed analysis of the Beck Depression Inventory is presented in Table 3.

**Table 3:** Distribution of responses to the Beck Depression Inventory.

**BDI interpretation**	**Stage 1 (*n =* 2,457) *N* (%)**	**Stage 2 (*n =* 1,626) *N* (%)**	**χ^2^**	Effect size^***^	***p***
Mean score	11.67 ± 9.47	13.76 ± 10.26		−0.211	<0.001^*^
No depression	1,232 (50.1)	674 (41.5)	527.871	0.359	<0.001^**^
Mild depression	765 (31.1)	540 (33.2)			
Moderate depression	232 (9.4)	175 (10.8)			
Severe depression	228 (9.3)	237 (14.6)			

* *U-Mann Whitney test*.

** *χ^2^ test*.

*** *d Cohen for U-Mann Whitney test and Fi/Cramer's V for χ^2^ test*.

**Figure 1:**
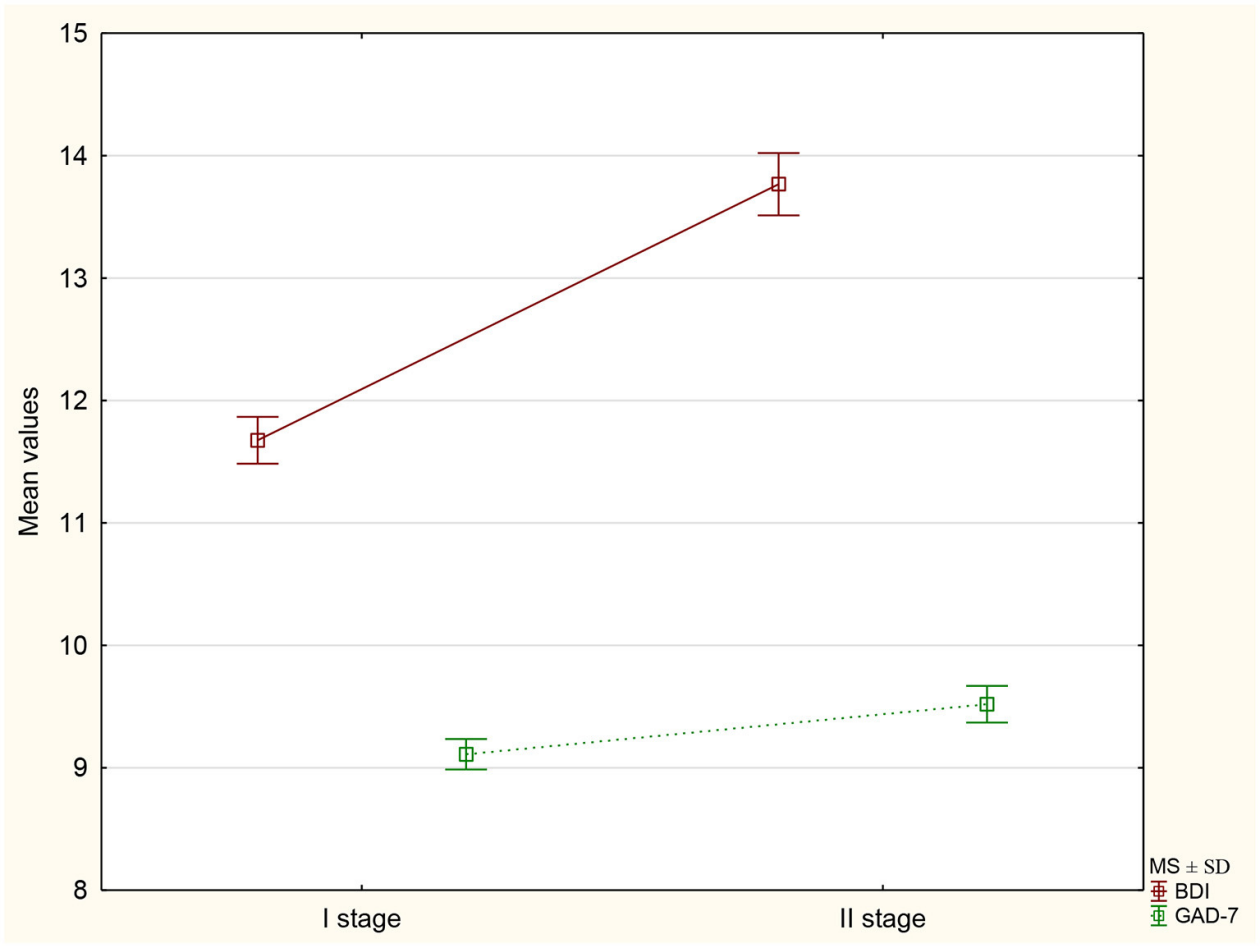
The mean values ± standard deviation of the BDI and GAD-7 scores at both stages of the study.

In the second stage of the study, the mean result was statistically significantly higher (by 2.09). The percentage of respondents whose final score indicates depressive disorders increased from 49.9 to 58.6%. There was a significant surge in the proportion of individuals who scored more than 26 points, indicating a risk of severe depression. The change in the mean values of the BDI and GAD-7 scales at both stages of the study is shown in Figure 1.

### The Assessment of the Quality of Life (Manchester Short Assessment of Quality of Life (MANSA)

A detailed comparative summary of individual MANSA questions is presented in Table 4.

**Table 4:** The level of satisfaction (including 3 of 7 answers which are positive: could not be better/I am satisfied/I am somewhat satisfied) for the individual questions of the MANSA scale by survey stage.

**Question**	Stage 1 (*n =* 2,457) *N* (%) M (SD)*	Stage 2 (*n =* 1,626) *N* (%) M (SD)*	**χ^2^**	Effect size^****^	***p***
Mean score*	60.65 ± 12.76	60.73 ± 12.27		−0.006	*p* = 0.821^**^
1. Are you generally satisfied with your life now?	1,379 (56.1)	856 (52.6)	4.785	0.034	0.028^***^
2. How satisfied are you with your job (other professional activities or schooling)?	1,323 (53.8)	786 (48.3)	11.881	0.053	<0.001^***^
3. Are you satisfied with your financial situation?	1,298 (52.8)	836 (51.4)	0,784	0.013	0.375^***^
4. Is there anyone you would consider a “close friend”?	1,835 (74.7)	1,406 (86.5)	83.022	0.142	<0.001^***^
5. Have you seen your friend this past week (visited/been visited; met outside of home or work)?	850 (34.6)	872 (53.6)	145.349	0.188	<0.001^***^
6. Are you satisfied with the number and quality of your friendships?	1,362 (55.4)	956 (58.8)	4.504	0.033	0.033^***^
7. How satisfied are you with your leisure activities (hobbies)?	1,165 (47.4)	723 (44.5)	3.426	0.029	0.064^***^
8. Are you satisfied with your housing situation?	1,583 (64.4)	1,097 (67.5)	4.004	0.031	0.045^***^
9. Do you have a sufficient sense of security?	1,555 (63.3)	1,157 (71.2)	27.156	0.081	<0.001^***^
10. Are you satisfied with your relationships with the people you live with?	1,734 (70.6)	1,198 (73.7)	4.656	0.034	0.031^***^
11. Are you satisfied with your sex life?	1,102 (44.9)	757 (46.6)	1.146	0.016	0.284^***^
12. How satisfied are you with your relationship with your family?	1,689 (68.7)	1,097 (67.5)	0.735	0.013	0.391^***^
13. Are you satisfied with your physical health?	1,284 (52.3)	766 (47.1**)**	10.378	0.050	0.001^***^
14. Are you satisfied with your mental health?	1,183 (48.1)	656 (40.3)	24.072	0.076	<0.001^***^

** *U-Mann Whitney test*.

*** *χ^2^ test*.

**** *d Cohen for U-Mann Whitney test and Fi / Cramer's V for χ^2^ test*.

*Significant effects (p < 0.05) are marked in bold*.

In terms of the MANSA scale, respondents completing the questionnaire at different stages of the pandemic did not show significant differences in the mean score. However, in the analysis of individual questions comprising the MANSA questionnaire, a statistically significant difference was observed in the percentage of respondents positively assessing their satisfaction with their life and work or school.

After <12 months of the pandemic, respondents were significantly more optimistic about their sense of security and their relationships with fellow household members and friends. However, a significantly lower satisfaction with their mental and physical health was observed.

### Influencing Factors

A detailed comparison of scores across stages against variables is shown in Table 5.

**Table 5:** Influence of sociodemographic, medical, and psychiatric history variables on individual scale scores in both stages of the study.

		**BDI**						**GAD-7**						**MANSA**					
		**Stage 1**	Effect size^**^	*p* ^*^	**Stage 2**	Effect size^**^	*p* ^*^	**Stage 1**	Effect size^**^	*p* ^*^	**Stage 2**	Effect size^**^	*p* ^*^	**Stage 1**	Effect size^**^	*p* ^*^	**Stage 2**	Effect size^**^	*p* ^*^
Sex	Male	10.5 ± 9.3	−0.148	** <0.001**	12.2 ± 10.0	−0.197	** <0.001**	7.3 ± 5.8	−0.366	** <0.001**	8.2 ± 6.2	−0.280	** <0.001**	60.8 ± 13.9	0.015	0.642	60.6 ± 12.8	−0.016	0.721
	Female	11.9 ± 9.5			14.2 ± 10.3			9.5 ± 6.2			9.9 ± 5.9			60.6 ± 12.6			60.8 ± 12.1		
Place of residence	Town/city	11.7 ± 9.5	0.021	0.772	13.7 ± 10.1	−0.028	0.941	9.1 ± 6.2	−0.016	0.594	9.5 ± 6.0	−0.016	0.846	60.7 ± 12.7	0.015	0.858	60.8 ± 12.1	0.015	0.977
	Rural area	11.5 ± 9.4			14.0 ±10.7			9.2 ± 6.2			9.6 ± 6.0			60.5 ± 12.9			60.6 ± 12.9		
Education	University education	11.1 ± 9.1	−0.303	** <0.001**	13.2 ± 10.1	−0.165	**0.002**	9.0 ± 6.1	−0.096	0.078	9.4 ± 6.1	−0.066	0.148	61.3 ± 12.4	0.266	** <0.001**	61.0 ± 12.2	0.065	0.171
	Other	14.1 ± 10.6			14.9 ± 10.4			9.6 ± 6.3			9.8 ± 5.9			57.8 ± 13.8			60.2 ± 12.3		
Relationship status	In a relationship	10.8 ± 8.8	−0.221	** <0.001**	12.4 ± 9.6	−0.227	** <0.001**	9.0 ± 6.2	−0.048	0.246	9.0 ± 6.1	−0.147	**0.003**	62.0 ± 12.6	0.237	** <0.001**	62.5 ± 12.4	0.237	** <0.001**
	Single	12.9 ± 10.1			14.7 ± 10.6			9.3 ± 6.1			9.9 ± 6.1			59.0 ± 12.7			59.6 ± 12.0		
Past psychiatric treatment	Yes	16.3 ± 11	0.587	** <0.001**	18.5 ± 11.7	0.560	** <0.001**	11.3 ± 5.9	0.466	** <0.001**	11.9 ± 5.8	0.508	** <0.001**	55.7 ± 12.8	−0.492	** <0.001**	56.7 ± 13.1	−0.407	** <0.001**
	No	10.5 ± 8.6			12.5 ± 9.6			8.5 ± 6.1			8.9 ± 6.0			61.9 ± 12.4			61.8 ± 11.9		
Recent suspicion of Covid-19	Yes	12.9 ± 9.5	0.136	0.163	14.5 ± 10.2	0.087	0.082	9.6 ± 6.1	0.081	0.441	10.2 ± 6.2	0.147	**0.021**	58.4 ± 13.2	−0.177	0.164	60.2 ± 11.7	−0.058	0.359
	No	11.6 ± 9.5			13.6 ± 10.3			9.1 ± 6.2			9.3 ± 6.0			60.7 ± 12.7			60.9 ± 12.4		
Recent mandatory quarantine	Yes	12 ± 10.5	0.030	0.831	14.8 ± 10.6	0.115	0.062	9.0 ± 6.6	−0.015	0.785	10.4 ± 6.3	0.178	**0.015**	60.2 ± 14.7	−0.036	0.741	59.4 ± 12.0	−0.131	0.063
	No	11.7 ± 9.4			13.6 ± 10.2			9.1 ± 6.2			9.3 ± 6.0			60.7 ± 12.7			61.0 ± 12.3		
COVID-19 diagnosis	Yes	15.4 ± 7.2	0.438	0.194	15.0 ± 10.9	0.123	0.142	7.6 ± 3.6	−0.295	0.717	10.5 ± 6.3	0.178	0.068	53.8 ± 11.7	−0.562	0.232	59.9 ± 12.5	−0.072	0.473
	No	11.7 ± 9.5			13.7 ± 10.2			9.1 ± 6.2			9.4 ± 6.0			60.7 ± 12.8			60.8 ± 12.2		
Covid-19 diagnosis in loved ones	Yes	12.2 ± 0.2	0.089	0.362	13.9 ± 10.4	0.048	0.441	10.1 ± 6.0	0.163	0.063	9.6 ± 6.0	0.050	0.313	61.5 ± 12.5	0.071	0.441	60.7 ± 11.9	−0.008	0.695
	No	11.6 ± 9.5			13.4 ± 10.1			9.1 ± 6.2			9.3± 6.0			60.6 ± 12.8			60.8 ± 12.8		
Search for	Yes	12.3 ± 9.3	0.178	** <0.001**	13.8 ± 9.8	0.000	0.432	9.7 ± 6.1	0.262	** <0.001**	9.8 ± 6.0	0.100	0.064	60.2 ± 12.6	−0.093	**0.011**	61.1 ± 11.7	0.057	0.417
Additional information on Covid-19	No	10.6 ± 9.7			13.8 ± 10.7			8.1 ± 6.1			9.2 ± 6.0			61.4 ± 13.0			60.4 ± 12.7		
Tracking daily Covid-19 statistics	Yes	12.5 ± 9.6	0.235	** <0.001**	13.9 ± 10.1	0.029	0.334	9.8 ± 6.2	0.311	** <0.001**	9.8 ± 6.0	0.100	**0.046**	60.2 ± 12.7	−0.101	**0.005**	60.9 ± 11.9	0.024	0.631
	No	10.3 ± 9.1			13.6 ± 10.4			7.9 ± 6.0			9.2 ± 6			61.5 ± 12.9			60.6 ± 12.6		
Loss of earning opportunities	Yes	14.1 ± 10.7	0.325	** <0.001**	18.1 +11.0	0.527	** <0.001**	10.1 ± 6.3	0.209	** <0.001**	11.5 ± 6.8	0.389	** <0.001**	57.0 ± 13.3	−0.373	** <0.001**	54.9 ± 12.3	−0.613	** <0.001**
	No	10.9 ± 8.9			12.6 ± 9.8			8.8 ± 6.1			9.0 ± 6.0			61.8 ± 12.4			62.3 ± 11.8		

* *U-Mann Whitney test*.

** *d Cohen*.

*Significant effects (p < 0.05) are marked in bold*.

In both stages, women had higher total BDI and GAD-7 scores than men. It was noted that respondents with university education had lower BDI scores than other respondents during both stages of the study. Loss of earning opportunities lowered scores on all scales and both stages of the analysis significantly, similarly to respondents with a history of mental issues. Searching for additional information on COVID-19 and tracking daily statistics correlated with scores on all scales; however, this relationship was only observed only in the first stage of the study.

### Correlation Between Scales

At each stage, a correlation was noted between BDI and GAD-7 total scores (stage 1: *r* = 0.7, *p* < 0.001; stage 2: *r* = 0.73, *p* < 0.001).

An inverse correlation was observed between GAD-7 and MANSA (stage 1: *r* = −0.51, *p* < 0.001; stage 2: *r* = −0.59, *p* < 0.001) and between BDI and MANSA in both stages of the study (stage 1: *r* = –0.63, *p* < 0.001; stage 2: *r* = –0.71, *p* < 0.001).

Age correlated very weakly with BDI (stage 1: *r* = –0.10, *p* < 0.001; stage 2: *r* = –0.14, *p* < 0.001), with GAD-7 (stage 1: *r* = −0.06, *p* = 0.005; stage 2: *r* = –0.12, *p* < 0.001) and MANSA (stage 1: −0.01, *p* = 0.67; stage 2: *r* = 0.05, *p* = 0.69).

### Search for Confounding Factors—Analysis of Covariance (ANCOVA)

The analysis of covariance showed a significant effect of reduced earning opportunities and tracking statistics on death and morbidity rates on BDI, GAD-7, and MANSA final scores. In addition, the impact of age was found to be significant in testing the ANCOVA model that included the total GAD-7 and MANSA scores. However, sex, education level or place of residence did not affect the differences in the scores on the scales in both stages of the study. A detailed summary of the analysis of covariance is shown in Table 6.

**Table 6:** Effects of confounding factors on differences between study stages (ANCOVA).

**Variable**	**BDI**		**GAD-7**		**MANSA**	
	**F**	***p***	**F**	***p***	**F**	***p***
Age	1.33	0.078	1.42	**0.038**	1.41	**0.041**
Sex	0.39	0.530	1.23	0.266	0.08	0.771
Education	2.05	0.152	0.05	0.808	6.39	**0.011**
Place of residence	0.65	0.584	0.05	0.987	0.48	0.699
Relationship status	0.001	0.998	2.09	0.148	0.04	0.834
Healthcare professional	3.628	0.057	3.95	0.047	0.04	0.849
Earning opportunities	8.65	**0.003**	5.86	**0.016**	7.30	**0.007**
Psychiatric/psychological treatment	0.04	0.852	0.01	0.914	1.54	0.215
Psychiatric medications	0.002	0.962	0.008	0.929	3.03	0.081
Testing for COVID-19	0.002	0.967	0.15	0.699	0.23	0.633
COVID-19 diagnosis	0.281	0.597	0.87	0.351	1.08	0.298
Covid-19 diagnosis in loved ones	0.013	0.910	1.11	0.293	0.57	0.452
Searching for information online	6.929	**0.009**	7.41	**0.007**	5.41	**0.02**
Tracking morbidity/death rates	8.154	**0.004**	10.834	**0.001**	3.72	**0.050**

*Significant effects (p < 0.05) are marked in bold*.

## Discussion

Observations to date clearly indicate a significant impact of the COVID-19 pandemic on the mental health of people worldwide (29). In the literature review to date, there are few reports comparing the mental health of people across its different waves. One of these is a longitudinal study conducted in the UK, which began on 21 March 2020 and involved weekly online data collection from participants. It facilitated the assessment of anxiety and depressive symptoms using the GAD-7 and PHQ-9 scales. Between 23 March and 9 August 2020, 70,000 volunteers participated in the study. Due to the lack of regular respondents, 36,520 persons ultimately met the participation criteria. The study showed a reduction in the severity of depressive and anxiety symptoms as the pandemic progressed, especially during the period of easing restrictions. In addition, it was confirmed that women, individuals with a lower level of education, single persons and those with a psychiatric condition were more likely to develop depressive and anxiety symptoms, especially during the first period of the pandemic (16). Similar observations were made in China. The study consisted of two measurements of psychotic disorders during the pandemic peak, which took place between 31 January and 9 February 2020. A repeat survey took place during the period of decline in the number of cases, i.e., from 15 to 28 March 2020. The results showed a reduction in anxiety and stress as the pandemic progressed but an increase in an increase indepressive symptoms (17).

An analogous observation was made in Spain, where a study took place after 2 and 5 weeks following the declaration of a state of emergency in the country when the number of infections and deaths due to COVID-19 was one of the highest in Europe.

A number of restrictions were imposed, including limiting movement only to necessary activities. In this study, the PHQ2, GAD-2, and PCL-C-2 scales were used to evaluate the mental health of respondents. An increase in the prevalence of depressive symptoms was demonstrated between the stages, but no statistically significant differences were found for anxiety and post-traumatic stress disorder (PTSD) (30). A study was also conducted in Austria, where the first stage of the study took place 4 weeks after introducing a lockdown, i.e., on 4 April 2020 and ended with the lifting of curfew-−30 April 2020. The repeat study took place 6 months after introducing the movement ban, i.e., between 7 and 21 September 2020. The number of participants in both stages was 437. Scales for depression (PHQ-9), anxiety (GAD-7), insomnia symptoms (ISI), quality of life (WHO-QoL BREF), well-being (WHO-5), and feelings of stress (PSS-10) were used to assess the mental health of respondents. As the pandemic progressed, there was a reduction in the severity of depressive and anxiety symptoms. In addition, there was an increase in satisfaction with one's well-being, and stress levels decreased (31). Observations reported in Germany also confirmed a reduction in the aforementioned symptoms (32). The results of our study are inconsistent with the above reports and indicate that, despite the passage of time, both depressive and anxiety symptoms, which were already at a high level at the initial stage, exceeded the reports from before the pandemic (16, 17, 30–33). There are many reasons for the observed differences. Firstly, the different stages of the severity of the pandemic in individual countries should be mentioned, which corresponded to the period of distribution of the questionnaire, which was associated with different levels of disease, mortality and applicable restrictions (20–22, 34). Moreover, comparable groups are not homogeneous in terms of gender, age and place of residence, which, as is known, may have an impact on the final result of the study. Undoubtedly, the cultural aspects, the level of social trust in both the authorities and health care workers, and the availability of their services, which during the period of significant intensification of the pandemic, varied in many countries also have a significant impact.

According to the report published in 2019 on mental health in the European Union, the prevalence of anxiety disorders in the Polish population was 3.9% and depressive disorders 5.1%. Moreover, it found that they occur more frequently in women than men (34).

Many factors may have contributed to exacerbated depressive and anxiety symptoms during the second wave, corresponding to the peak of the pandemic in Poland. One of them is undoubtedly the data collection period. In the early stages, COVID-19 incidence and death rates were significantly lower than at the pandemic peak, when they culminated in Poland (35). COVID-19 statistics occupied most of the media coverage. The Specter of total lockdown was looming over the nation. As is well known, incoming information regarding the increasing number of new outbreaks and deaths is closely correlated with mental health (36), which was also confirmed in our study. A similar phenomenon was also observed in other countries. According to reports from India, the media constantly reporting the progress of the pandemic abroad contributed to a massive increase in anxiety and fear among the population (37).

Due to the significant spread of SARS-CoV-2, after a brief period of laxity, the country's government decided to maintain and even tighten restrictions, which resulted in a substantial reduction in the entertainment of all kinds and contributed to spending more leisure time at home (23). Introducing restrictions in a short time hindered the healthy adaptation of people to the new reality, intensifying their feelings of anxiety (38). This hypothesis is also supported by analyzing individual items of the MANSA scale, where respondents assessed subjective satisfaction with their life, work and school significantly lower, potentially leading to increased negative emotions (39). The reason for this may be the prolongation of remote forms of teaching/work, which limit social interactions. Furthermore, it should be noted that it is essential for young people entering adulthood to interact with their peer group, expand their autonomy and reduce their ties with their parents (39), which are significantly reduced in the current situation.

An additional stress factor is the lack of prospects and ability to estimate when life will return to its pre-pandemic state, making it impossible to plan for the future (38).

On the other hand, the initial stage of the pandemic, despite the lower incidence among patients, concerned the first wave; the novelty of the pandemic situation could have a significant impact on mental health. Therefore, we believe that the cumulative effect of the pandemic threat had a considerable influence on the mental condition of respondents.

Although our study did not show significant statistical differences in the overall assessment of the quality of life, the individual components of quality of life differed substantially. In the repeat survey, the respondents' satisfaction with both mental and physical health significantly decreased. The reduction in activity makes it impossible to take care of oneself in the same way as before the pandemic (38). The closure of gyms and the autumn/winter season and the consequent decrease in physical activity may contribute to our health deterioration (40). A review article published by Paluska and Schwenk confirmed that aerobic exercise or strength training contributes significantly to reducing depressive symptoms. In addition, meditation and relaxation are beneficial in reducing anxiety levels (41).

The repeat survey showed a higher level of feelings of security among respondents, increased the frequency of encounters with loved ones, and paradoxically, despite the rise in infections, a lower level of anxiety over contracting the disease. At the same time, however, the level of adherence to government recommendations and interest in searching for covid-19 information online declined, which significantly affected the final results of the survey. This may be because people started adapting to a new reality as the pandemic continues. Studies of isolated people indicate that the process of getting used to new conditions is inevitable; there is no alternative to it, only the way can change (42). Moreover, as time passes, the virus becomes more and more familiar.

Our study results showed that single people and those living in a town or city are more prone to depressive disorders. Loss of earning opportunities is another possible cause of increased depressive and anxiety symptoms and decreased life satisfaction. According to a study conducted by Freeman, deterioration of social and economic conditions is one of the factors exacerbating depressive symptoms (43). Therefore, the economic downturn has a significant impact on our mental health, contributing to increased suicides, which was confirmed in studies conducted before the pandemic (44, 45).

The authors are aware of the limitations of this study, which is undoubtedly the lack of representativeness of the study group in relation to Polish society. The predominance of females, the average age of respondents, the number of people living in town and cities and with university education may influence the final result of the observation, especially because previous reports show that young age and female sex increase the risk of escalation of depressive and anxiety symptoms (16). It is related to the psychological resilience of older individuals resulting from greater life experience, habituation to solitude and better ability to control emotions (45, 46). It should also be stressed that a significant limitation of the study is an unbalanced sociodemographic distribution of respondents at both stages of the study in terms of age, relationship status and education, which may significantly interfere with the final result. The analysis of covariance (ANCOVA) showed a statistically significant influence of age on the GAD-7 and MANSA scale values and the level of education on the MANSA scale. Another methodological limitation is the data collection method in the form of an anonymous survey distributed through a social media portal. As a result, the authors had no way of verifying the number of people who started but did not complete the survey or the number of people who knew about the survey. Due to the nature of the work (complete anonymity and the way the questionnaire was distributed), the authors of this report could not provide psychological support to respondents. Nevertheless, one can hope that participation in the study prompted the participants to take a closer look at their mental health and, if necessary, seek medical assistance. Although the authors distributed the questionnaire within the same groups on the social networking site, no assessment was made of whether the same respondents participated in both stages of the study, therefore the results should be treated as a comparison of the two groups.

The Covid-19 pandemic is a human experience with significant impacts on our physical and mental health (38). Support is critical during this time. Providing training in stress management could contribute to reducing anxiety levels among the population (36).

The physical distance, social isolation and quarantine resulting from the many restrictions increased loneliness among the population, which has many negative clinical implications that include attention, concentration and affect disorders (47). Furthermore, according to scientific reports, the intensified stress during the pandemic also increased alcohol and tobacco consumption (48). Analysis of the above variables showed that further observation of representative groups is needed to assess the impact of the COVID-19 pandemic on population health and to develop mental health support programmes for those most in need as soon as possible.

## Conclusions

The ongoing COVID-19 pandemic affects the mental and physical health of the Polish population. Women, individuals with lower education levels and those living in towns and cities show a greater severity of both anxiety and depression symptoms. Furthermore, economic instability significantly affects mental health by leading to anxiety and depressive symptoms and reduced quality of life. Considering the results of this study and the results reported by other authors, there is an urgent need to develop and implement mental health support programmes widely available to those who need them during the pandemic.

## Data Availability Statement

The raw data supporting the conclusions of this article will be made available by the authors, without undue reservation.

## Ethics Statement

The studies involving human participants were reviewed and approved by Bioethics Committee of the Wroclaw Medical University, Poland (approval number: KB-471/2020). The patients/participants provided their written informed consent to participate in this study.

## Author Contributions

MB and AM-M: conceptualization and methodology. MB: software, data curation, and project administration. MB, AM-M, KK, and BB: validation, writing—original draft preparation, writing—review and editing, and visualization. MB and KK: formal analysis. MB, KK, and BB: investigation. BB and MB: resources. AM-M: supervision and funding acquisition. All authors have read and agreed to the published version of the manuscript.

## Funding

The presented research was carried out on the subject according to the records in the Simple system with the number SUB.C290.21.010.

## Conflict of Interest

The authors declare that the research was conducted in the absence of any commercial or financial relationships that could be construed as a potential conflict of interest.

## Publisher's Note

All claims expressed in this article are solely those of the authors and do not necessarily represent those of their affiliated organizations, or those of the publisher, the editors and the reviewers. Any product that may be evaluated in this article, or claim that may be made by its manufacturer, is not guaranteed or endorsed by the publisher.
